# Antiplatelets and Anticoagulants in Vitreoretinal Surgery: A Systematic Review

**DOI:** 10.3390/life13061362

**Published:** 2023-06-09

**Authors:** Filippo Confalonieri, Vanessa Ferraro, Alessandra Di Maria, Alessandro Gaeta, Josè Luis Vallejo-Garcia, Paolo Vinciguerra, Xhevat Lumi, Goran Petrovski

**Affiliations:** 1 Department of Ophthalmology, IRCCS Humanitas Research Hospital, Via Manzoni 56, Rozzano, 20089 Milan, Italy; vanessa.ferraro@humanitas.it (V.F.); alessandra.di_maria@humanitas.it (A.D.M.); jose_luis.vallejo_garcia@humanitas.it (J.L.V.-G.); paolo.vinciguerra@hunimed.eu (P.V.); 2 Department of Biomedical Sciences, Humanitas University, Via Rita Levi Montalcini 4, Pieve Emanuele, 20090 Milan, Italy; 3 Center for Eye Research and Innovative Diagnostics, Department of Ophthalmology, Institute for Clinical Medicine, University of Oslo, Kirkeveien 166, 0450 Oslo, Norway; xhevat.lumi@kclj.si; 4 Department of Ophthalmology, Oslo University Hospital, Kirkeveien 166, 0450 Oslo, Norway; 5 Department of Internal Medicine and Medical Specialties (DIMI), Università di Genova, Viale Benedetto XV, 6, 16132 Genova, Italy; alessandro.gaeta01@gmail.com; 6 Eye Hospital, University Medical Centre Ljubljana, Zaloška Cesta 2, 1000 Ljubljana, Slovenia; 7 Department of Ophthalmology, University of Split School of Medicine and University Hospital Centre, 21000 Split, Croatia

**Keywords:** vitreoretinal surgery, surgical technique, antiplatelet, anticoagulant, eye surgery

## Abstract

**Background**: Antiplatelets and anticoagulants have substantially influenced contemporary vitreoretinal surgical practices. The availability of new oral blood thinners has recently spurred a renewed interest in the clinical approach to vitreoretinal surgical conditions since it may be difficult for the surgeon to collect sufficient evidence-based data to decide whether to discontinue or continue such medications. **Materials and Methods**: We conducted a systematic review on the use of antiplatelets and/or anticoagulants in the perioperative setting in vitreoretinal surgery and their possible complications, following the Preferred Reporting Items for Systematic Reviews and Meta-Analyses (PRISMA) guidelines. The level of evidence, according to the Oxford Centre for Evidence-Based Medicine (OCEM) 2011 guidelines, and the quality of evidence, according to the Grading of Recommendations Assessment, Development, and Evaluation (GRADE) system, were assessed for all included articles. **Results**: In total, 2310 articles were initially extracted, out of which 1839 articles were obtained after duplicates were removed and their abstracts were screened. A total of 27 articles were included in the full-text review. Finally, a remaining 22 articles fulfilled the inclusion criteria. **Conclusions**: Even though there is just a small number of studies with solid results, the advantage of using antiplatelets and/or anticoagulants in vitreoretinal surgery seems to outweigh the disadvantages, which are mainly related to postoperative hemorrhagic complications.

## 1. Introduction

In recent years, treatment with antiplatelet and anticoagulant drugs has become highly prevalent in the elderly population [1,2,3,4,5]. Likewise, the advent of minimally invasive vitreoretinal surgical devices increased the range of surgically treatable vitreoretinal diseases and, thus, the number of patients suitable for surgery [6,7].

No large prospective, randomized clinical trials (RCTs) have been conducted on the relative risk of using antiplatelets and/or anticoagulants perioperatively in vitreoretinal surgery; still, a high amount of variability exists among eye departments on preoperative drug management, especially regarding novel anticoagulants [8].

Evidence has recently been raised as to the risk of cardiovascular accidents related to the discontinuation of blood thinners before surgery; meanwhile, the addition of low-dose unfractionated heparin bridging may have an insufficient effect on reducing thromboembolic events, thus raising the risk of severe bleeding [9].

In this systematic review, we analyze the available literature on the use of antiplatelet and/or anticoagulant drugs in patients undergoing vitreoretinal surgery with the aim to draw evidence-based conclusions on the best practices for these patient groups in the perioperative period.

## 2. Materials and Methods

A systematic review was conducted to explore the risks and complications associated with the use of antiplatelets and/or anticoagulants in patients undergoing vitreoretinal surgery. The review followed the Preferred Reporting Items for Systematic Reviews and Meta-Analyses (PRISMA) guidelines, which provide a framework for reporting systematic reviews in a transparent and comprehensive manner [10]. Although the review protocol was not recorded in the study design, a registration number was available for consultation. This indicates that this study was registered with a relevant authority or database, which helps improve transparency and reduce bias in the research process. To identify relevant articles, a systematic literature search was performed on 30 December 2022 using controlled vocabulary and test words for “vitreoretinal surgery”, “vitrectomy”, “retina”, “retinal diseases’’, “antiplatelet”, and “anticoagulant” and specific keywords for new anticoagulant agents (NAO). The search was conducted in electronic databases, including Ovid Medline, Embase (Ovid), Cochrane Register of Controlled Trials, and Cochrane Database of Systematic Reviews. There were no restrictions on language, publication type, study design, or publication date. The complete search strategy is given in Appendix A.

Additionally, the reference lists of the identified articles were manually examined to identify any potentially relevant studies that may have been missed by the electronic searches. This process helped ensure comprehensive coverage of the available literature. After the preparation of the list of all electronic data, three reviewers (FC, VF, and AG) examined the titles and abstracts independently and identified relevant articles. The inclusion criteria included studies that investigated the administration of antiplatelets and/or anticoagulants in patients undergoing vitreoretinal surgery and assessed the risk of bleeding-related complications. The exclusion criteria encompassed review studies, pilot studies, case series with fewer than 12 patients, case reports, photo essays, and studies written in languages other than English. Animal studies, cadaveric studies, and studies involving pediatric patients were also excluded. The same reviewers then reviewed the full texts of the selected articles to confirm their eligibility based on the inclusion and exclusion criteria. Any disagreements among the reviewers were resolved through consensus; experienced reviewers (ADM and JLVG) were consulted when needed to provide additional expertise. No unpublished data were obtained from the corresponding authors of the selected articles, suggesting that the analysis was conducted based on the available published information. The level of evidence was assessed according to the Oxford Centre for Evidence-Based Medicine (OCEM) 2011 guidelines [11], which provide a framework for evaluating the strength and quality of evidence in medical research. The quality of the evidence was evaluated using the Grading of Recommendations Assessment, Development, and Evaluation (GRADE) system [12], which helps assess the certainty of evidence and make recommendations. Overall, the systematic review followed rigorous methods outlined by the PRISMA guidelines and utilized a comprehensive search strategy to identify relevant studies. The inclusion and exclusion criteria were clearly defined and the level and quality of the evidence were assessed using established frameworks.

## 3. Results

**Figure 1:**
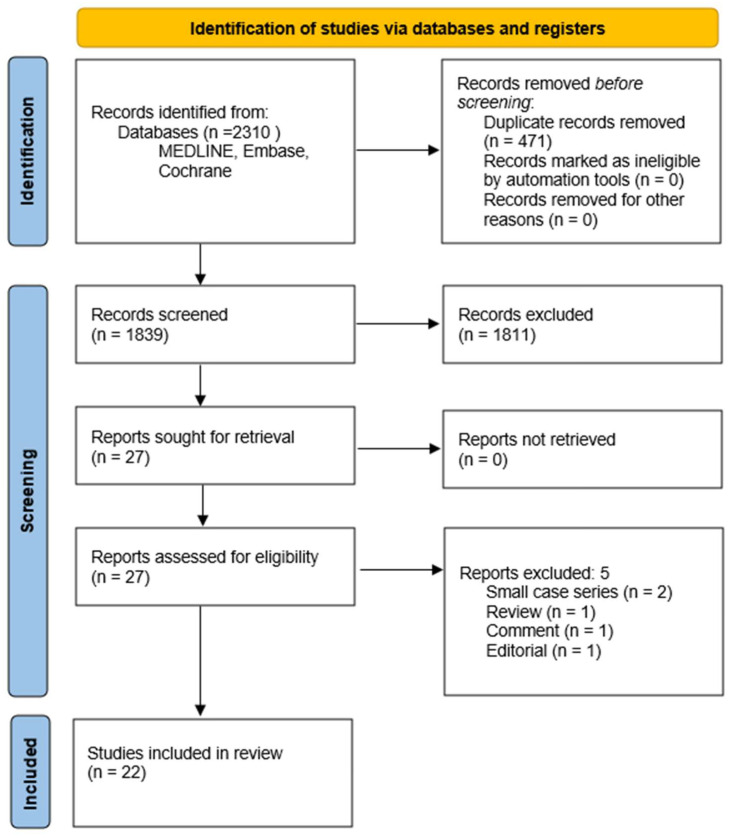
Flow diagram of this study according to Preferred Reporting Items for Systematic Reviews and Meta-Analyses (PRIMA) guidelines [10].

Figure 1 summarizes the flow diagram of the search approach; the results of the analysis are collected and displayed in Table 1. A total of 2310 articles were extracted. After removing duplicates and screening the abstracts, 1839 articles remained for further evaluation. Subsequently, 27 articles were selected for a full-text review to assess their eligibility based on the specific inclusion/exclusion criteria. Ultimately, 22 articles met all of the inclusion criteria and were included in the analysis. Appendix B provides a summary of the reasons behind each article’s selection.

No data synthesis could be achieved due to the heterogeneity of the available data and the research designs. Therefore, the present systematic review offers a qualitative analysis that is conducted in a chronological and systematic manner below.

Narendran et al. [34], in 2003, examined the medical information of 541 consecutive patients having their vitreoretinal surgery prospectively examined. They recorded the anticoagulant status and, in the trial, seven individuals received warfarin and sixty patients received aspirin. A total of eleven incidences of choroidal hemorrhage were reported and one of those cases was a warfarin user. Warfarin’s link to bleeding was statistically significant (relative risk 6.185). They concluded that warfarin was linked to problems with bleeding in the perioperative period after vitreoretinal surgery. They suggested that stopping aspirin before surgery has no role in reducing the bleeding complications; however, if the patient’s thromboembolic risk is low, warfarin may be withdrawn.

Dayani et al. [33], in 2006, wanted to assess the risk of hemorrhagic complications linked to vitreoretinal surgery in individuals whose warfarin medication was continued throughout the surgical period. They included patients whose international normalized ratio (INR) was elevated above normal while undergoing vitreoretinal procedures and 1737 consecutive charts were reviewed. After categorizing the patients according to the INR value, they concluded that there was no hemorrhagic risk during the procedure, per se, and the increased postoperative hemorrhage rate was represented by self-limiting episodes with no need for intervention.

Fu et al. [32], in 2007, aimed at describing the clinical course of patients undergoing a vitreoretinal surgery who received warfarin at the same time; this included 25 patients involved in being followed-ups. Cerebrovascular illness, artificial heart valves, deep vein thrombosis, atrial fibrillation, and hypercoagulable condition were among the indications for anticoagulation. The follow-ups lasted from four months to three years (median, 19.5 months). The range for the INR was from 1.5 to 3.1 (median, 2.0). The range of postoperative visual acuity was from 20/20 to 20/400 (median, 20/100). One patient suffered a subretinal hemorrhage during surgery that was connected to the external drainage of subretinal fluid and scleral buckling. No other patients experienced any intraoperative problems. They concluded that for individuals undergoing vitreoretinal surgery who are taking warfarin, successful visual and anatomical outcomes are possible.

Brown et al. [31], in 2011, aimed at evaluating the risk of perioperative hemorrhage in diabetic vitrectomies. The included patients were those on anticoagulants or antiplatelets affected by tractional macular detachment, premacular hemorrhage, and/or non-clearing vitreous hemorrhage secondary to diabetes mellitus (DM). The study was a retrospective, comparative cohort analysis of all patients who underwent a diabetic pars plana vitrectomy (PPV) conducted by a single surgeon over a 30-month period. A total of 97 eyes were enrolled in the analysis. Throughout the procedure, 27 eyes remained on anticoagulation. Anticoagulation-related perioperative problems were nonexistent. The frequency of postoperative vitreous hemorrhages or surgical revisions did not differ between the two groups. They concluded that anticoagulation or antiplatelet medications do not increase the incidence of intraoperative or postoperative vitreous hemorrhage in diabetic patients undergoing PPV. To prevent problems brought on by their systemic condition, anticoagulants and antiplatelets may be safely maintained during surgery.

Chandra et al. [30] looked at a group of 60 patients who were on warfarin treatment and receiving PPV. The participants involved in this retrospective case-control study were compared to another group of 60 patients who had identical presenting symptoms. Additionally, an online poll was conducted to gauge UK practice. In total, 2% of the patients included were on warfarin with an INR ranging from 0.94 to 4.6. (median 2.3). While none occurred in the warfarin group, there were two occurrences of suprachoroidal hemorrhages in the control group. In the warfarin group, 12 patients with rhegmatogenous retinal detachment (RRD) had vitreous hemorrhages compared to four in the control group. Based on the INR, 48 responders (81%) said they would recommend that patients stop taking warfarin before vitreoretinal surgery. They concluded that patients who continued taking warfarin did not experience a higher complication rate than the controls; however, patients with RRD who were on warfarin had a higher risk of having vitreous hemorrhage upon presentation.

El-Sanhouri et al. [29], in 2011, aimed at determining the rate of retinal tears after posterior vitreous detachment (PVD) and vitreous hemorrhage in patients taking systemic anticoagulants. A total of 260 eyes with acute PVD and vitreous hemorrhages were included in the act of looking for any signs of retinal tear or detachment. Those who were using systemic anticoagulants and those who were not were separated into individual groups. A total of 137 study subjects (53%) were taking anticoagulants, compared to 123 (47%) who were not. Overall, 46% of the patients using anticoagulants exhibited evidence of a retinal tear, compared to 72% of the individuals who were not taking any anticoagulants. Moreover, 23% of those on any anticoagulants experienced an RRD, compared to 37% of individuals not taking any anticoagulants. They concluded that patients with vitreous hemorrhage and acute PVD taking anticoagulants have a lower risk of retinal tears.

Fabinyi et al. [28], in 2011, aimed at assessing the impact of preoperative anticoagulation and antiplatelet medication on postoperative vitreous cavity bleeding after PPV for diabetic eye disease. To do so, they included 139 patients whose clinical records were retrospectively reviewed. Anticoagulation or antiplatelet medications were being used in 68 of the 155 (43.9%) eyes of 139 patients at the time of surgery. A total of 29 (42.6%) patients were receiving treatment at the time of surgery. Postoperatively, eight of these patients (27.6%) experienced considerable vitreous hemorrhage, with four (13.8%) requiring further surgery; in total, thirty-nine patients (57.4%) had stopped their medication before surgery and, of them, three (7.7%) required further surgery, while four (10.3%) experienced recurrent bleeding. None of the 87 patients (6.9%) with postoperative vitreous hemorrhages that required no anticoagulation or antiplatelet medication required further surgery. Patients who were on anticoagulant or antiplatelet medication at the time of surgery had an increased risk of developing persistent bleeding and needing a second surgery (OR = 4.8, *p* = 0.0045, and OR = 6.6, *p* = 0.024, respectively). They concluded that patients on anticoagulants or antiplatelet therapy requiring surgery for diabetic eye diseases are at increased risk of persistent postoperative vitreous cavity hemorrhage and necessitate rePPV at a higher rate. This risk seems to be decreased by appropriate preoperative treatment discontinuation.

Mason et al. [27], in 2011, aimed at analyzing the hemorrhagic complication rate in patients on warfarin therapy, clopidogrel therapy, or neither receiving 25-gauge PPV. In the warfarin group, there were 64 eyes; meanwhile, in the clopidogrel group, there were 125 eyes. By using the INR, patients taking warfarin were separated into four groups; meanwhile, 110 eyes who were not taking warfarin or clopidogrel made up the control group. The reasons for anticoagulant medication were various and included thromboembolic disease (16%), valvular heart disease (17%), and atrial fibrillation (38%). Cardiovascular stent (49%), coronary artery bypass grafting (24%), and a history of transient ischemic stroke (16%) were the most frequent reasons for antiplatelet treatment. The peribulbar or retrobulbar block did not cause any anesthesia-related hemorrhagic problems in any of the patients. One (1.6%) of the 64 PPV in the warfarin group (*p* = 0.6531), five (3.7%) of the 136 PPV procedures in the clopidogrel group (*p* = 1.0), and four (3.6%) of the 110 PPV procedures in the control group experienced transient vitreous hemorrhage. No patient experienced choroidal or retrobulbar hemorrhages. They concluded that patients on systemic anticoagulation or platelet inhibitor medication have a very low incidence of 25-gauge PPV hemorrhagic sequelae; it was advised that patients stick with their therapy regimen given the hazards involved with quitting these medicines.

Malik et al. [26], in 2012, documented the results of PPV under subconjunctival anesthesia without stopping anticoagulant and antiplatelet medications preoperatively. The documentation included anatomical outcomes, adequate analgesia, visual acuity, and intraoperative and postoperative bleeding. A total of 56 eyes that received 63 PPVs were included. A total of thirty-two patients were using aspirin and clopidogrel, eighteen were on warfarin, five were taking clopidogrel, three were taking both, two were taking acetylsalicylic acid and dipyridamole, and one was taking both medications. Every patient received adequate analgesia. There were no issues throughout the surgery; however, a surgical vitreous hemorrhage affected seven eyes (13%). Most of the patients (79%) had improved postoperative visual acuity, 16% had stable visual acuity, and 5% had worsened visual acuity at the most recent follow-up visit. They concluded that PPV is feasible in patients for whom delaying ocular surgical surgery would be risky or in whom stopping anticoagulants is medically contraindicated.

Passemard et al. [25], in 2012, aimed at assessing the complication rate in peribulbar anesthesia in patients receiving oral anticoagulation. In a single academic setting, they carried out a retrospective case series study. The anticoagulants were not discontinued before surgery. Three groups of patients were created: patients who did not receive any anticoagulant or antiplatelet therapy; patients who received anticoagulant therapy; and those who received aspirin, clopidogrel, or both. In total, 206 patients and 236 of their eyes were included. Across the groups, there was no significant difference in the frequency of severe or moderate postoperative hemorrhagic complications. Nevertheless, patients using antiplatelet medications experienced potentially blinding hemorrhagic complications more frequently (*p* = 0.003). They concluded that patients using anticoagulants can probably receive peribulbar anesthesia for vitreoretinal surgery without risk and that patients receiving antiplatelet medications experience serious bleeding problems more frequently.

Ryan et al. [24], in 2013, prospectively analyzed a cohort of patients who did not suspend antiplatelet or anticoagulant treatment preoperatively, looking for the risk of bleeding after vitreoretinal surgery. A total of 107 PPVs on 85 patients were included in the study and the intra- and post-operative bleeding complications were recorded. Proliferative diabetic retinopathy (PDR) and DM were the two most important independent predictors of intraoperative bleeding and postoperative bleeding, respectively. They concluded that there were no cases of significant postoperative choroidal bleeding or uncontrolled intraoperative hemorrhages. It was typical to experience postoperative vitreous cavity bleeding, as well as mild hemorrhage, following a vitrectomy. Most individuals taking antiplatelet or anticoagulant medication can do so without risk throughout the perioperative period of vitreoretinal surgery.

Witmer et al. [23], in 2013, analyzed if, in eyes with acute, posterior vitreous detachment, oral anticoagulation changed the relationship between vitreous hemorrhages (VH) and retinal tears. The final retrospective analysis comprised 336 eligible eyes. In total, 118 (35%) of the eyes experienced vitreous hemorrhages, with 43% of the patients using aspirin, clopidogrel, or warfarin, compared to 31% not taking these drugs (*p* = 0.03). In individuals with VH, retinal tears occurred in 46% of cases, compared to 27% in patients without VH (*p* = 0.0007). Compared to the 52% of patients not using aspirin, clopidogrel, or warfarin, 39% of the VH patients on these drugs experienced retinal tears. They concluded that patients with symptoms of acute PVD and a vitreous hemorrhage frequently have retinal tears (46%); when an acute PVD occurs, patients taking aspirin, clopidogrel, or warfarin are more likely to present with a vitreous hemorrhage.

Brillat et al. [22], in 2015, aimed at evaluating the hemorrhagic risk of aspirin medication during the perioperative period of RRD surgical repair. This case-control study comprised 322 patients who underwent RRD surgery with (*n* = 74) or without (*n* = 248) an anterior segment, choroidal, intravitreal, and/or subretinal hemorrhage. Trauma history, vitreoretinal surgery, diabetic retinopathy, and the use of clopidogrel and/or vitamin K antagonists were the exclusion criteria. Aspirin was not substantially linked to postoperative or intraoperative bleeding problems (*p* = 0.8). In 47% of the instances, scleral buckling (with cryotherapy and gas tamponade) occurred and in 53% of the cases, pars plana vitrectomy was performed. A decreased single-operation anatomical success rate (74% vs. 84%, *p* = 0.03) was linked to the bleeding problems. Moreover, there was a tendency to create a link between the final visual acuity, a higher overall rate of RRD recurrences, and bleeding complications. They concluded that aspirin does not significantly increase the risk of hemorrhagic complications during or following RRD repair surgery and that the rate of single-operation anatomic success is lowered as a result of perioperative bleeding.

Grand et al. [21], in 2016, aimed at assessing patients receiving systemic therapy with new oral anticoagulants (NOAs) and antiplatelet medications, such as rivaroxaban, apixaban, dabigatran, and prasugrel. The objective was to assess the incidence and type of perioperative hemorrhagic complications related to vitreoretinal surgery. To do so, a retrospective analysis of a group of patients who received any vitreoretinal surgery over a two-year period while being treated with anticoagulant and antiplatelet medications was carried out and 36 eyes from 33 individuals were found to have undergone vitreoretinal surgery while receiving systemic anticoagulant and antiplatelet treatment. No eyes had subretinal, suprachoroidal, or retrobulbar bleeding as postoperative sequelae. After postoperative vitreous cavity bleeding in four eyes (11.1%), two eyes (5.5%) needed additional surgical intervention while two eyes (5.5%) resolved on their own. They concluded that patients may successfully undergo vitreoretinal surgery while maintaining medication with rivaroxaban, apixaban, dabigatran, and prasugrel.

Ajudani et al. [20], in 2017, aimed to assess the prevalence of ocular hemorrhages in patients on aspirin undergoing 20-gauge PPV for PDR versus patients who did not use aspirin. The two groups consisted of ninety patients each. There was bleeding in 56 patients. The incidence of bleeding did not differ significantly between the aspirin (33 patients) and control groups (23 patients) (*p* = 0.1). Moreover, there were no discernible changes in the kind of bleeding between the two groups (*p* = 0.11). Age, gender, hypertension, surgery type, and laboratory results did not differ significantly between patients who had bleeding and those who did not. They concluded that, compared to the control group, taking aspirin is not linked to an increased risk of bleeding after PPV. Therefore, the medication does not need to be stopped immediately in diabetic individuals undergoing PPV.

Meillon et al. [19], in 2018, aimed at assessing the hemorrhagic complications of vitreoretinal surgeries, which were carried out in seven ophthalmologic facilities, on patients receiving or not receiving antiplatelet or anticoagulant medicines. During surgery and for a month after, the patients’ characteristics, surgical methods, and complications were noted. They examined 844 cases, of which 148 received antiplatelet treatment, 63 received anticoagulant treatment, and 18 received NOAs treatment; 53 patients (6.6%) experienced one or more hemorrhagic problems in one eye. In contrast to antiplatelets, anticoagulant drugs were not linked to hemorrhagic complications in a univariate analysis. They concluded that neither antiplatelet nor anticoagulant drugs were linked to hemorrhagic complications in the perioperative period of vitreoretinal surgery; the continuation of these therapies should be taken into consideration.

Andonegui et al. [18], in 2019, made the first protocol of a RCT to assess the differences in perioperative complications of PPV between individuals who receive anticoagulant treatment before surgery and those who do not. This trial’s results will offer novel data about the potential for anticoagulant treatment continuation during PPV. The results of the trial are not yet available. 

Bemme et al. [17], in 2020, aimed at exploring the risk of perioperative bleeding in the perioperative period of surgery for retinal detachment in patients taking antiplatelets or anticoagulants versus those who do not take them. To do so, they investigated the rate of all perioperative hemorrhages during PPV; scleral buckling, with or without drainage; or combined procedures in patients receiving various types of anticoagulation, such as acetylsalicylic acetate (ASA), clopidogrel, heparin, low molecular weight heparin, and phenprocoumon. The study comprised 893 individuals with primary RRD: 192 on anticoagulation and 701 as controls who did not receive anticoagulation. The results showed no statistically significant increase in perioperative hemorrhages with ASA 100 mg, nor phenprocoumon, compared to the controls. However, the frequency of bleeding complications varied significantly depending on the surgical procedure: scleral buckling plus drainage had the highest rates of hemorrhages compared to scleral buckling without drainage and PPV. Additionally, the most prevalent kind of perioperative hemorrhage was subretinal bleeding. They concluded that there is no need to stop ASA medication, nor Vitamin K antagonist anticoagulants with an INR, in the subtherapeutic range prior to vitreoretinal surgery. 

Louison et al. [16], in 2020, aimed to analyze the risk of hemorrhagic complications in elective macular surgery between patients who were on anticoagulants versus those who were not. They collected data on patient characteristics, surgical techniques, hemorrhagic complications, and antithrombotic statuses through e-case report forms, before and after vitreoretinal surgery. Patients with retinal detachment, PDR, and previous retinal hemorrhage were excluded. A total of 748 procedures were analyzed and, at the time of surgery, 202 of these patients (27%) received antithrombotic therapy, 146 (19.5%) received antiplatelet agents, and 47 (6.3%) received anticoagulants (*n* = 47). In all, 92 patients (12.3%) experienced one or more hemorrhagic complications, with the non-antithrombotic group accounting for 63 (11.5%) of these cases and the antithrombotic group for 29 (14.4%) of these cases, respectively. There was no difference between the therapy groups in terms of ocular bleeding problems. They concluded that most patients taking antiplatelets or anticoagulants may continue taking them without risk during macular surgery.

Lauerman et al. [15], in 2021, assessed the causes, risk factors, and potential effects of serious bleeding issues after vitreoretinal surgery, as well as investigating the potential effects of antiplatelet and anticoagulant medications. To do so, they performed a prospective clinical trial that included scleral buckling and PPV while creating a standardized categorization to rate the level of bleeding. In addition, the impact of known systemic illnesses before surgery, the kind of anesthetic used, the type of surgery performed, the intraoperative blood pressure, and the use or modification of antiplatelet or anticoagulant drugs were all examined in relation to intraoperative bleeding. Data were gathered from 374 eyes. They discovered that serious bleeding episodes were substantially linked with concurrent conditions, such as DM and carotid artery stenosis, the presence of diabetic retinopathy, younger age, and scleral buckling accompanied by a transscleral puncture. On the other hand, serious intraoperative bleeding episodes were not significantly affected by the administration of antiplatelets, anticoagulant drugs, or both. They concluded that there was no evidence of an increased bleeding risk associated with concurrent antiplatelets, anticoagulant medication use, or both. 

Starr et al. [14], in 2021, gathered data on hemorrhagic complications in patients undergoing vitreoretinal surgery while being on NOAs or not taking any anticoagulants. To do so, the electronic health records of patients receiving PPV who did not have a history of ocular bleeding were analyzed across many centers over time. In total, 2956 (5.1%) of the 58,131 eyes that underwent PPV had taken anticoagulant medication before the procedure. A single eye among every 828 eyes (1.4%) experienced a surgical hemorrhage. In total, 50 of every 2956 (1.29%) eyes that had previously used anticoagulation experienced a hemorrhage, compared to 778 of every 55,175 (1.41%) of the eyes that had not. They concluded that using NOAs does not seem to be linked to a higher risk of postoperative intraocular bleeding.

Guise et al. [13], in 2022, aimed to gather a consensus on the opportunity of avoiding non-vitamin K oral anticoagulants (NOAs). A total of 291 patients were included in the study as they were taking these medications while having cataract surgery or vitreoretinal surgery, mostly under sub-Tenon’s anesthesia. There were no complications that could have resulted in the loss of vision in the immediate postoperative period; however, two patients with vitreoretinal diseases (3.8%) experienced moderate hemorrhagic complications on day five and two patients with cataract diseases (0.8%) experienced minor hemorrhagic complications on days one and fourteen after surgery. Three patients (1% of the total number of cataract patients) experienced a mild thromboembolic event within the first thirty days after surgery while taking their NOA. They concluded that the continued use of NAOs for patients undergoing cataract and vitreoretinal surgery brings along a negligible risk of hemorrhagic complications.

## 4. Discussion

A step forward towards better management regarding patients on anticoagulant and antiplatelet medication in vitreoretinal surgery can be achieved by a clear understanding of the potential complications, both ocular and systemic, arising from the practice of stopping or continuing the use of such drugs [8].

The correct management of anticoagulants and antiplatelets in vitreoretinal surgery allows for minimizing the risk of perioperative and intraoperative ocular hemorrhages, on the one hand, while protecting from cardiovascular accidents that may coincide with the physically stressful time of surgery, on the other [35,36,37]. Furthermore, delaying the surgery in some urgent cases, such as macula-on retinal detachments, to allow for an adequate time of recovery from the drugs may lead to a worse functional and anatomical prognosis [38]. 

As the results of this systematic review are highly diverse, the following dissertation is trying to derive evidence from the collection of all the qualitatively evaluated papers, first categorizing the studies according to the type of surgery or anesthesia and then by the type of antiplatelet or anticoagulant drugs used, the papers that showed common conclusions, the types of complications, and the study design.

Mason et al. and Passemard et al. [25,27] investigated the hemorrhagic complication rate in patients receiving retrobulbar and peribulbar anesthesia while on only anticoagulants or on antiplatelets and anticoagulants, respectively, versus no therapy. The conclusions are encouraging as they show a low risk of complications and high safety regarding local anesthetic injections; although, Passemard et al. found a slightly increased bleeding risk in patients on antiplatelets [25]. Even though it is well known that retrobulbar hemorrhages can lead to dreadful functional impairments up to blindness [39], these studies show that it may be reasonable not to delay the surgery as the complication rate is the same if the patient is on anticoagulants, or it is only slightly increased if the patient is on antiplatelets.

Narendran et al. [34] were the first to investigate the association between aspirin and warfarin intake and the risk of bleeding complications, showing no increased risk for aspirin but a slightly increased risk in the perioperative period for warfarin. This was confirmed by Dayani et al. [33], who specified that warfarin-associated bleeding risk is actually self-limiting and does not need intervention; it is therefore safe to continue taking warfarin before vitreoretinal surgery. The safety of warfarin in vitreoretinal surgery was confirmed also by Fu et al. [32], who also introduced the concept of relative safety for scleral buckling. The high-safety profile of aspirin on both scleral buckle and PPV was also confirmed by Brillat et al., while Bemme et al. also confirmed anticoagulants’ safety in terms of scleral buckle surgery [22]. Brown et al. further expanded the concept of anticoagulation and antiplatelet maintenance in neovascular conditions, such as diabetic vitrectomies, showing no increased bleeding risk [31]. On the contrary, Fabinyi et al. [28] showed that both anticoagulants and antiplatelets lead potentially to a hemorrhage that requires a second surgery to be resolved [28]. Chandra et al. [30] confirmed the safety of continuing warfarin in patients requiring PPV but added that the presentation of these patients’ RRDs is more often accompanied by vitreous hemorrhages. The same applies to the increased chance of vitreous hemorrhages in PVD and retinal tear presentation for patients on anticoagulants and antiplatelets, as shown by Witmer et al. [23]. El-Sanhouri et al. [29] expanded on that by showing anticoagulation to even be a protective factor; patients with vitreous hemorrhages and acute PVD taking anticoagulants were shown to have a lower risk of retinal tears. A plethora of further studies confirmed that it is safe continue, and possibly inappropriate to stop, taking anticoagulants and antiplatelets, especially in the face of an increased risk of systemic thromboembolic accidents [16,17,20,22,24,26,27].

More recently, the same results on the effects of warfarin and aspirin in terms of vitreoretinal surgery were confirmed by authors who tested the latest oral anticoagulants and antiplatelets. Grand et al. [21] were the first to show that patients may successfully undergo vitreoretinal surgery while maintaining medications, such as rivaroxaban, apixaban, dabigatran, and prasugrel. The same results on the safety of not stopping NOAs were demonstrated afterward by various authors [13,14,19].

Our systematic review has certain limitations that should be acknowledged. Firstly, due to our focus on articles published in English, we may have excluded valuable studies published in other languages, leading to a potential language bias. Secondly, the analyzed articles exhibited considerable heterogeneity, making direct comparisons of the results and the production of a meta-analysis challenging. This heterogeneity arises from variations in the drugs used, the types of bleeding complications evaluated, the studied populations, the analyzed vitreoretinal conditions, the surgeons’ experience, and the surgical techniques employed. 

A complex issue in drawing conclusions is the fact that it is difficult, if not impossible, to create completely comparable groups of patients in terms of systemic conditions, INR values, drug dosages, eye pathology, and surgical approach and technique while at the same time considering iatrogenic-induced trauma. Furthermore, more elements of complexity should be added in consideration of the fact that INR can vary a lot in its range before generating certain complications.

In general, the recent literature as a whole on antiplatelets and anticoagulants in vitreoretinal surgery is showing that there is no considerable increase in the complication rate in patients who are continuing the blood thinning agents they are on and, on the contrary, it is becoming evident that stopping these drugs may lead to dreadful systemic complications. The sentiment that there is no need for stopping these agents is increasingly becoming a part of common practice.

## 5. Conclusions

Despite the fact that there is only one completed RCT showing the safety of performing vitreoretinal surgery on patients on anticoagulants and antiplatelets [15], the literature is nearly unanimously in recommending that patients continue taking antiplatelets and/or anticoagulants. All the evidence is directed towards showing that this practice does not increase the risk of hemorrhagic complications that need a second surgery, both in PPV and scleral buckle. On the contrary, it is advantageous due to it reducing the time between the diagnosis and surgery and maintaining high protection from cardioembolic accidents.

## Figures and Tables

**Table 1:** Characteristics, quality, and level of evidence of the included studies and the type of antiplatelets/anticoagulants utilized in the included studies.

Author (et al.)	Year	Study Design	Study Sample (Eyes)	Type of Intervention	Type of Antiplatelet and/or Anticoagulant	Grade	Level
Guise [13]	2022	Prospective	52	Sub/tenon-s block, vitreoretinal surgery	Dabigatran, rivaroxaban and apixaban	Low	4
Starr MR [14]	2021	Retrospective	58,131	Vitreoretinal surgery	Direct oral anticoagulants	Low	4
Lauermann P [15]	2021	Prospective	374	Pars plana vitrectomy and scleral buckling	All	Low	4
Louison S [16]	2020	Prospective	748	Pars plana vitrectomy	Aspirin and clopidogrel, warfarin and fluindione, rivaroxaban, apixaban and dabigatran, heparin	Low	4
Bemme S [17]	2020	Retrospective	893	Pars plana vitrectomy and scleral buckling	Aspirin and clopidogrel, heparin, phenprocoumon	Very low	4
Andonegui J [18]	2019	Prospective	96	Pars plana vitrectomy +/− phacoemulsification or scleral buckling	dabigatran, rivaroxaban, apixaban or edoxaban, acenocumarol	Moderate	2
Meillon C [19]	2018	Prospective	804	Pars plana vitrectomy +/− scleral buckling	Aspirin and clopidogrel, warfarin, new oral anticoagulants	Low	3
Ajudani R [20]	2017	Prospective	180	20-gauge vitrectomy	Aspirin	Low	3
Grand MG [21]	2016	Retrospective	36	Pars plana vitrectomy +/− scleral buckling	Rivaroxaban, apixaban, dabigatran etexilate, or prasugrel	Very low	4
Brillat E [22]	2015	Prospective	322	ars plana vitrectomy +/− scleral buckling	Aspirin	Low	4
Witmer MT [23]	2013	Retrospective	336	Spontaneous PVD	Aspirin, Clopidogrel, Warfarin	Very low	4
Ryan A [24]	2013	Prospective	107	Pars plana vitrectomy +/− scleral buckling	Aspirin, clopidogrel, warfarin	Low	3
Passemard M [25]	2012	Retrospective	206	Pars plana vitrectomy +/− phacoemulsification	Warfarin, fluindione, and acenocoumarol, aspirin, clopidogrel	Very low	4
Malik AI [26]	2012	Retrospective	56	Pars plana vitrectomy +/− peeling +/− pars plana lensectomy	ASA, clopidogrel, warfarin, dipyridamole	Very low	4
Mason JO III [27]	2011	Retrospective	299	Pars plana vitrectomy	Clopidogrel, warfarin	Very low	4
Fabinyi DC [28]	2011	Retrospective	155	Pars plana vitrectomy	ASA, clopidogrel, warfarin, dipyridamole	Very low	4
El-Sanhouri AA [29]	2011	Prospective	260	Acute PVD and VH	Warfarin, ASA, NSAIDs, clopidogrel, and pentoxiphylline	Low	3
Chandra A [30]	2011	Retrospective	120	Pars plana vitrectomy	Warfarin	Very low	4
Brown JS [31]	2011	Retrospective	97	Pars plana vitrectomy	Aspirin, warfarin, clopidogrel	Very low	4
Fu AD [32]	2007	Retrospective	25	Pars plana vitrectomy	Warfarin	Very low	4
Dayani PN [33]	2006	Retrospective	54	Pars plana vitrectomy +/− scleral buckling +/− scleral buckling	Warfarin	Very low	4
Narendran N [34]	2003	Prospective	541	Pars plana vitrectomy and external cryo/ buckle	Aspirin, warfarin	Low	3

## Data Availability

Data are available on reasonable request by the corresponding author.

## Appendix A

Documentation of literature search.

Research question: Antiplatelets and anticoagulants in vitreoretinal surgery.

The following databases were searched:

**Database**	**Number of Retrieved References**
MEDLINE (Ovid):	832
Embase (Ovid):	1264
Cochrane Central Register of Controlled Trials:	214
Number of references before deduplication:	2310
Number of references after deduplication:	1839

Search syntax:

**Ovid-Databases**	
exp/	Exploded index term
/	After an index term indicates a subject heading was selected.
.ti,ab,kf.	Search for a term in the title, abstract, and author keywords
.kw.	=keyword heading
*	At the end of a term indicates that this term has been truncated, diet* retrieves both diet, diets, dietary.
Adj3	Search for two terms next to each other, in any order, up to 3 words in between.
**Cochrane Library**	
ti,ab,kw	Search for a word in the title, abstract, or keyword
NEAR/3	Search for two terms next to each other, in any order, up to 3 words in between.

Ovid MEDLINE(R) ALL 1946 to 30 December 2022.

**#**	**Searches**	**Results**
1	vitreoretinal surgery/or vitrectomy/	15,994
2	exp Retina/su [Surgery]	3225
3	exp Retinal Diseases/su [Surgery]	22,714
4	(((vitreoretinal or vitreo retinal or retina or macula*) adj3 (surg* or operat* or procedure* or repair or translocation* or incision*)) or retinotom* or vitrectom* or (vitreous adj2 resection*)).ti,ab,kf.	22,474
5	Retina/	80,738
6	Retinal Perforations/	5726
7	((retina* or macula*) adj3 (perforation* or hole* or tear* or break* or rupture)).ti,ab,kf.	9348
8	Retinal Detachment/	20,805
9	(retinal detachment* or ablatio retinae or amotio retinae or detached retina or pigment epithelial detachment* or pigment epithelium detachment* or retinal pigment epithelial detachment* or retinal pigment epithelium detachment* or retina ablatio* or retinal ablation* or retina separation or retinal separation or retina shrinkage or retina sublation* or sublatio retinae or rhegmatogenous retinal detachment or RPE detachment* or separated retina).ti,ab,kf.	23,654
10	or/1-9	130,509
11	exp Anticoagulants/	240,000
12	exp Platelet Aggregation Inhibitors/	134,248
13	exp Fibrinolytic Agents/	182,304
14	(anticoagulant* or anti coagulant* or anti coagulating or anticoagulation or anticoagulative* or antithrombotic* or anti-thrombotic* or antithrombocytic* or anti-thrombocytic* or antiplatelet* or anti-platelet* or antiaggreg* or anti aggreg* or (platelet adj2 (inhibitor* or antagonist*)) or ((thrombocyte or thromboxane) adj2 (inhibit* or antagonist*))).ti,ab,kf.	162,262
15	Prasugrel Hydrochloride/or prasugrel*.ti,ab,kf.	2950
16	exp Heparin Antagonists/or exp Heparin/or (heparin* or UFH or ariven or arteven or calcilean or hepalean or hepathrom or leparan or lipo hepin or liquaemin or liquemin or pabyrin or pularin or thromboliquin* or thrombophlogat or thrombophob or vetren or clexan* or klexan* or lovenox or fragmin or dalteparin or beparine or uniparin or clarin or contusol or disebrin or eleparon or elheparin or elheparon or epiheparin or helberina or hep lock or hepaflex or hepalean or heparina* or heparitin or hepflush or hepsal or inviclot or lipohepin or menaven or monoparin or mucoitin polysul* or multiparin or nevparin or noparin or panheparin or panhepin or panheprin or parinix or praecivenin or pularin or thrombareduct or thromboreduct or thrombosamine or vetren or vr 496 or vr496 or antixarin or danaproid or danaparoid or enoxaparin* or glycosaminoglycan* or nadroparin* or mesoglycan* or tedelparin* or certoparin or tinzaparin or parnaparin or dalteparin or reviparin or fraxiparin* or danaparoid or lomoparan or “org 10,172” or mesoglycan or pentosan polysul* or dermatan sul* or heparan sul* or sp54 or sp 54 or cy222 or cy 222 or cy216 or cy 216).ti,ab,kf.	150,881
17	exp Heparin, Low-Molecular-Weight/or (LMH or LMHs or LMWH or LMWHs or bm 2123 or bm2123 or choay or ebpm 1 or ebpm 2 or ebpm 3 or ebpm1 or ebpm2 or ebpm3 or ff 1034 or ff1034 or fr 860 or fr860 or gag 869 or “pk 007” or sandoz 6700 or traxyparine or embolex or monoembolex or certoparin or danaparoid or parnaparin or kb101 or fluxum or lohepa or lowhepa).ti,ab,kf.	17,117
18	exp Warfarin/or (warfarin or warfarine or warfarinum or uniwarfin or waran or warfar or warfil 5 or warfilone or warnerin or warfant or acetonylbenzylhydroxycoumarin or adoisine or aldocumar or antrombin* or athrombin* or befarin or carfin or circuvit or compound 42 or coumadan or coumadin* or coumafene or coumaphene or dagonal or jantoven or kumatox or maforan or marevan or orfarin or panwarfarin or panwarfin or prothromadin or sofarin or tintorane or warf compound 42 or (vitamin adj2 antagonist*) or antivitamin k or VKA or AVK or phenindione or Sinthrome or nicoumalone or phenprocoumon or Marcoumar or Marcumar or Falithrom or phenprocoumon* or aldocumar or carfin or kumatox or lawarin or prothromadin or sofarin or tedicumar or tintorane or coumadin* or coumarin* or cumarin* or phenprocoum* or phenprocum* or dicoumar* or dicumar* or acenocoumar* or acenocumar* or fluindione or phenindione or clorindione or diphenadione or ethyl biscoumacetate).ti,ab,kf.	59,599
19	exp Aspirin/or (aspirin* or acetyl salicylic acid* or acetyl?salicylic acid* or acetylsalicylic acid* or clopidogrel* or 2 chlorophenyl or clopilet or grepid or inhiplat or iscover or mdco 157 or mdco157 or myogrel or osvix or pcr 4099 or pcr4099 or plavitor or Plavix or pm 103 or pm103 or pregrel or sr 25,989 or sr 25990c or sr25989 or zopya or zylagren or zyllt or ARC1779 or AZD6140 or alprostadil or asasantin or carnitine or cilostazol or clopidogrel or cloricromene or defibrotide or dilazep or dipyridamol* or disintegrin* or ditazol or E5880 or E5510 or epoprostenol* or fluribrofen or iloprost* or indobufen or isbogrel or ketanserin* or ketoprofen or ketorolac or levamisol* or ligustrazine* or tromethamine* or milrinone* or mopidamol* or naudicelle or nimesulide or ozagrel* or phthalzinol or picotamide or policosanol or prasugrel or procainamide or sarpogrelate or satigrel or sulphinpyrazone or sulfinpyrazone or suloctadil or terutroban or ticagrelor or ticlopidine or trapidil or triflusal or vorapaxar or oky046 or “oky 046” or oky 1581 or kbt3022 or kbt 3022 or fut 175 or cv4151 or cv 4151 or acylpyrin or aloxiprimum or colfarit or dispril or easprin or ecotrin or endosprin or magnecyl or micristin or polopirin or polopiryna or r16co5y76e or solprin or solupsan or zorprin).ti,ab,kf.	149,470
20	(ticlopidine* or ticagrelor* or abciximab* or eptifibatide* or tirofiban* or cilostazol* or dipyridamole* or (FX* adj4 (antag* or inhib* or block*)) or (Factor X* adj4 (antag* or inhib* or block*)) or dabigatran* or pradaxa or rendix or rivaroxaban or apixaban* or edoxaban* or betrixaban* or anisindione or apolate sodium or beciparcil or chlorophacinone or defibrotide or dextran sulfate or diphenadione or fluindione or ghilanten or glycerophosphoinositol inositolphosphodiesterase or glycosaminoglycan polysulfate or iliparcil or inclacumab or ljp 1082 or low molecular weight dextran sulfate or mopidamol or naroparcil or phenindione or ru 45,703 or tecarfarin or torapsel or tretoquinol or tretoquinol derivative or uproleselan or lomoparan or orgaran or apixaban or rivaroxaban or xarelto or edoxaban or lixiana or etexilate or agatroban or xabans).ti,ab,kf.	44,563
21	or/11–20	632,271
22	10 and 21	2030
23	exp animals/or exp animal experimentation/or exp animal experiment/or exp models animal/or nonhuman/or exp vertebrate/or exp vertebrates/or exp Cadaver/	26,040,272
24	exp humans/or exp human experimentation/or exp human experiment/	20,951,986
25	23 not 24	5,088,914
26	(veterinary or animal or animals or cadaver* or rabbit or rabbits or rodent or rodents or rat or rats or mouse or mice or rabbit or rabbits or pig or pigs or porcine or pigeon* or horse* or equine or cow or cows or cattle or bovine or goat or goats or donkey* or sheep or ovine or dog or dogs or canine or feline or dolphin* or beetle* or fish or fishes or zebrafish* or bluefish*).ti.	2,416,882
27	limit 22 to “all child (0 to 18 years)”	116
28	25 or 26 or 27	5,572,969
29	22 not 28	1260
30	limit 29 to (english language and yr = “2002-Current”)	832

Search history link (may require log in): https://ovidsp.ovid.com/ovidweb.cgi?T=JS&NEWS=N&PAGE=main&SHAREDSEARCHID=3cEsuvZZy2fRTH67ItSJKEWt7SHwGhtlLzYhZdoC4ian7hkCC2BHDS32HDA7R1L4A (accessed on 30 December 2022).

Embase Classic + Embase 1947 to 30 December 2022.

**#**	**Searches**	**Results**
1	vitreoretinal surgery/or exp vitrectomy/or retina surgery/or exp retina detachment surgery/	34,895
2	exp retina/su [Surgery]	859
3	exp retina disease/su [Surgery]	26,371
4	(((vitreoretinal or vitreo retinal or retina or macula*) adj3 (surg* or operat* or procedure* or repair or translocation* or incision*)) or retinotom* or vitrectom* or (vitreous adj2 resection*)).ti,ab,kf.	27,794
5	retina/or retina tear/	80,822
6	((retina* or macula*) adj3 (perforation* or hole* or tear* or break* or rupture)).ti,ab,kf.	12,289
7	retina detachment/	39,781
8	(retinal detachment* or ablatio retinae or amotio retinae or detached retina or pigment epithelial detachment* or pigment epithelium detachment* or retinal pigment epithelial detachment* or retinal pigment epithelium detachment* or retina ablatio* or retinal ablation* or retina separation or retinal separation or retina shrinkage or retina sublation* or sublatio retinae or rhegmatogenous retinal detachment or RPE detachment* or separated retina).ti,ab,kf.	30,858
9	retina macula hole/	6350
10	(retina macula degeneration or macula hole* or macular hole* or macula lutea hole*).ti,ab,kf.	6743
11	or/1–10	152,170
12	anticoagulant agent/	138,737
13	antithrombocytic agent/	52,077
14	(anticoagulant* or anti coagulant* or anti coagulating or anticoagulation or anticoagulative* or antithrombotic* or anti-thrombotic* or antithrombocytic* or anti-thrombocytic* or antiplatelet* or anti-platelet* or antiaggreg* or anti aggreg* or (platelet adj2 (inhibitor* or antagonist*)) or ((thrombocyte or thromboxane) adj2 (inhibit* or antagonist*))).ti,ab,kf.	263,502
15	exp prasugrel/or prasugrel*.ti,ab,kf.	11,143
16	exp heparin/or (heparin* or UFH or ariven or arteven or calcilean or hepalean or hepathrom or leparan or lipo hepin or liquaemin or liquemin or pabyrin or pularin or thromboliquin* or thrombophlogat or thrombophob or vetren or clexan* or klexan* or lovenox or fragmin or dalteparin or beparine or uniparin or clarin or contusol or disebrin or eleparon or elheparin or elheparon or epiheparin or helberina or hep lock or hepaflex or hepalean or heparina* or heparitin or hepflush or hepsal or inviclot or lipohepin or menaven or monoparin or mucoitin polysul* or multiparin or nevparin or noparin or panheparin or panhepin or panheprin or parinix or praecivenin or pularin or thrombareduct or thromboreduct or thrombosamine or vetren or vr 496 or vr496 or antixarin or danaproid or danaparoid or enoxaparin* or glycosaminoglycan* or nadroparin* or mesoglycan* or tedelparin* or certoparin or tinzaparin or parnaparin or dalteparin or reviparin or fraxiparin* or danaparoid or lomoparan or “org 10,172” or mesoglycan or pentosan polysul* or dermatan sul* or heparan sul* or sp54 or sp 54 or cy222 or cy 222 or cy216 or cy 216).ti,ab,kf.	271,416
17	exp low molecular weight heparin/or (LMH or LMHs or LMWH or LMWHs or bm 2123 or bm2123 or choay or ebpm 1 or ebpm 2 or ebpm 3 or ebpm1 or ebpm2 or ebpm3 or ff 1034 or ff1034 or fr 860 or fr860 or gag 869 or “pk 007” or sandoz 6700 or traxyparine or embolex or monoembolex or certoparin or danaparoid or parnaparin or kb101 or fluxum or lohepa or lowhepa).ti,ab,kf.	81,273
18	exp warfarin/or (warfarin or warfarine or warfarinum or uniwarfin or waran or warfar or warfil 5 or warfilone or warnerin or warfant or acetonylbenzylhydroxycoumarin or adoisine or aldocumar or antrombin* or athrombin* or befarin or carfin or circuvit or compound 42 or coumadan or coumadin* or coumafene or coumaphene or dagonal or jantoven or kumatox or maforan or marevan or orfarin or panwarfarin or panwarfin or prothromadin or sofarin or tintorane or warf compound 42 or (vitamin adj2 antagonist*) or antivitamin k or VKA or AVK or phenindione or Sinthrome or nicoumalone or phenprocoumon or Marcoumar or Marcumar or Falithrom or phenprocoumon* or aldocumar or carfin or kumatox or lawarin or prothromadin or sofarin or tedicumar or tintorane or coumadin* or coumarin* or cumarin* or phenprocoum* or phenprocum* or dicoumar* or dicumar* or acenocoumar* or acenocumar* or fluindione or phenindione or clorindione or diphenadione or ethyl biscoumacetate).ti,ab,kf.	144,607
19	exp acetylsalicylic acid/or (aspirin* or acetyl salicylic acid* or acetyl?salicylic acid* or acetylsalicylic acid* or clopidogrel* or 2 chlorophenyl or clopilet or grepid or inhiplat or iscover or mdco 157 or mdco157 or myogrel or osvix or pcr 4099 or pcr4099 or plavitor or Plavix or pm 103 or pm103 or pregrel or sr 25,989 or sr 25990c or sr25989 or zopya or zylagren or zyllt or ARC1779 or AZD6140 or alprostadil or asasantin or carnitine or cilostazol or clopidogrel or cloricromene or defibrotide or dilazep or dipyridamol* or disintegrin* or ditazol or E5880 or E5510 or epoprostenol* or fluribrofen or iloprost* or indobufen or isbogrel or ketanserin* or ketoprofen or ketorolac or levamisol* or ligustrazine* or tromethamine* or milrinone* or mopidamol* or naudicelle or nimesulide or ozagrel* or phthalzinol or picotamide or policosanol or prasugrel or procainamide or sarpogrelate or satigrel or sulphinpyrazone or sulfinpyrazone or suloctadil or terutroban or ticagrelor or ticlopidine or trapidil or triflusal or vorapaxar or oky046 or “oky 046” or oky 1581 or kbt3022 or kbt 3022 or fut 175 or cv4151 or cv 4151 or acylpyrin or aloxiprimum or colfarit or dispril or easprin or ecotrin or endosprin or magnecyl or micristin or polopirin or polopiryna or r16co5y76e or solprin or solupsan or zorprin).ti,ab,kf.	355,039
20	(ticlopidine* or ticagrelor* or abciximab* or eptifibatide* or tirofiban* or cilostazol* or dipyridamole* or (FX* adj4 (antag* or inhib* or block*)) or (Factor X* adj4 (antag* or inhib* or block*)) or dabigatran* or pradaxa or rendix or rivaroxaban or apixaban* or edoxaban* or betrixaban* or anisindione or apolate sodium or beciparcil or chlorophacinone or defibrotide or dextran sulfate or diphenadione or fluindione or ghilanten or glycerophosphoinositol inositolphosphodiesterase or glycosaminoglycan polysulfate or iliparcil or inclacumab or ljp 1082 or low molecular weight dextran sulfate or mopidamol or naroparcil or phenindione or ru 45,703 or tecarfarin or torapsel or tretoquinol or tretoquinol derivative or uproleselan or lomoparan or orgaran or apixaban or rivaroxaban or xarelto or edoxaban or lixiana or etexilate or agatroban or xabans).ti,ab,kf.	71,129
21	or/12–20	880,817
22	11 and 21	2355
23	(exp animal/or exp animal model/or nonhuman/) not exp human/	7,761,218
24	(cadaver* or veterinary or animal or animals or rabbit or rabbits or rodent or rodents or rat or rats or mouse or mice or rabbit or rabbits or pig or pigs or porcine or pigeon* or horse* or equine or cow or cows or cattle or bovine or goat or goats or donkey* or sheep or ovine or dog or dogs or canine or feline or dolphin* or beetle* or fish or fishes or zebrafish* or bluefish*).ti.	2,924,004
25	limit 22 to child	74
26	or/23–25	8,214,068
27	22 not 26	1878
28	limit 27 to (english and yr = “2002-Current”)	1264

Search history link (may require log in): https://ovidsp.ovid.com/ovidweb.cgi?T=JS&NEWS=N&PAGE=main&SHAREDSEARCHID=2jww9B63kxQNASok69A8ZvFJz1MOCifuAuH1zQ7eEDucVBrIqSncDGcJVINhvh40P (accessed on 30 December 2022).

**The Cochrane Library**:

#1	MeSH descriptor: [Vitreoretinal Surgery] explode all trees	37
#2	MeSH descriptor: [Vitrectomy] explode all trees	572
#3	MeSH descriptor: [Retina] explode all trees and with qualifier(s): [surgery—SU]	140
#4	MeSH descriptor: [Retinal Diseases] explode all trees and with qualifier(s): [surgery—SU]	1003
#5	((((vitreoretinal or “vitreo retinal” or retina or macula*) NEAR/3 (surg* or operat* or procedure* or repair or translocation* or incision*)) or retinotom* or vitrectom* or (vitreous NEAR/2 resection*))):ti,ab,kw (Word variations have been searched)	2728
#6	MeSH descriptor: [Retinal Perforations] explode all trees	240
#7	(((retina* or macula*) NEAR/3 (perforation* or hole* or tear* or break* or rupture))):ti,ab,kw (Word variations have been searched)	834
#8	MeSH descriptor: [Retina] this term only	679
#9	MeSH descriptor: [Retinal Detachment] explode all trees	374
#10	(retinal detachment* or ablatio retinae or amotio retinae or detached retina or pigment epithelial detachment* or pigment epithelium detachment* or retinal pigment epithelial detachment* or retinal pigment epithelium detachment* or retina ablatio* or retinal ablation* or retina separation or retinal separation or retina shrinkage or retina sublation* or sublatio retinae or rhegmatogenous retinal detachment or “RPE detachment” or separated retina):ti,ab,kw (Word variations have been searched)	2106
#11	{OR #1–#10}	4919
#12	MeSH descriptor: [Anticoagulants] explode all trees	5052
#13	MeSH descriptor: [Platelet Aggregation Inhibitors] explode all trees	4267
#14	MeSH descriptor: [Fibrinolytic Agents] explode all trees	2358
#15	((anticoagulant* or anti coagulant* or anti coagulating or anticoagulation or anticoagulative* or antithrombotic* or anti-thrombotic* or antithrombocytic* or anti-thrombocytic* or antiplatelet* or anti-platelet* or antiaggreg* or anti aggreg* or (platelet NEAR/2 (inhibitor* or antagonist*)) or ((thrombocyte or thromboxane) NEAR/2 (inhibit* or antagonist*)))):ti,ab,kw (Word variations have been searched)	27,656
#16	MeSH descriptor: [Prasugrel Hydrochloride] explode all trees	492
#17	(prasugrel*):ti,ab,kw (Word variations have been searched)	1256
#18	MeSH descriptor: [Heparin Antagonists] explode all trees	69
#19	MeSH descriptor: [Heparin] explode all trees	5033
#20	(heparin* or UFH or ariven or arteven or calcilean or hepalean or hepathrom or leparan or lipo hepin or liquaemin or liquemin or pabyrin or pularin or thromboliquin* or thrombophlogat or thrombophob or vetren or clexan* or klexan* or lovenox or fragmin or dalteparin or beparine or uniparin or clarin or contusol or disebrin or eleparon or elheparin or elheparon or epiheparin or helberina or hep lock or hepaflex or hepalean or heparina* or heparitin or hepflush or hepsal or inviclot or lipohepin or menaven or monoparin or mucoitin polysul* or multiparin or nevparin or noparin or panheparin or panhepin or panheprin or parinix or praecivenin or pularin or thrombareduct or thromboreduct or thrombosamine or vetren or vr 496 or vr496 or antixarin or danaproid or danaparoid or enoxaparin* or glycosaminoglycan* or nadroparin* or mesoglycan* or tedelparin* or certoparin or tinzaparin or parnaparin or dalteparin or reviparin or fraxiparin* or danaparoid or lomoparan or “org 10,172” or mesoglycan or pentosan polysul* or dermatan sul* or heparan sul* or sp54 or sp 54 or cy222 or cy 222 or cy216 or cy 216):ti,ab,kw (Word variations have been searched)	16,173
#21	MeSH descriptor: [Heparin, Low-Molecular-Weight] explode all trees	2087
#22	(LMH or LMHs or LMWH or LMWHs or bm 2123 or bm2123 or choay or ebpm 1 or ebpm 2 or ebpm 3 or ebpm1 or ebpm2 or ebpm3 or ff 1034 or ff1034 or fr 860 or fr860 or gag 869 or “pk 007” or sandoz 6700 or traxyparine or embolex or monoembolex or certoparin or danaparoid or parnaparin or kb101 or fluxum or lohepa or lowhepa):ti,ab,kw (Word variations have been searched)	1843
#23	MeSH descriptor: [Warfarin] explode all trees	1800
#24	(warfarin or warfarine or warfarinum or uniwarfin or waran or warfar or warfil 5 or warfilone or warnerin or warfant or acetonylbenzylhydroxycoumarin or adoisine or aldocumar or antrombin* or athrombin* or befarin or carfin or circuvit or compound 42 or coumadan or coumadin* or coumafene or coumaphene or dagonal or jantoven or kumatox or maforan or marevan or orfarin or panwarfarin or panwarfin or prothromadin or sofarin or tintorane or warf compound 42 or (vitamin adj2 antagonist*) or antivitamin k or VKA or AVK or phenindione or Sinthrome or nicoumalone or phenprocoumon or Marcoumar or Marcumar or Falithrom or phenprocoumon* or aldocumar or carfin or kumatox or lawarin or prothromadin or sofarin or tedicumar or tintorane or coumadin* or coumarin* or cumarin* or phenprocoum* or phenprocum* or dicoumar* or dicumar* or acenocoumar* or acenocumar* or fluindione or phenindione or clorindione or diphenadione or ethyl biscoumacetate):ti,ab,kw (Word variations have been searched)	11,736
#25	MeSH descriptor: [Aspirin] explode all trees	6294
#26	(aspirin* or acetyl salicylic acid* or acetyl?salicylic acid* or acetylsalicylic acid* or clopidogrel* or 2 chlorophenyl or clopilet or grepid or inhiplat or iscover or mdco 157 or mdco157 or myogrel or osvix or pcr 4099 or pcr4099 or plavitor or Plavix or pm 103 or pm103 or pregrel or sr 25,989 or sr 25990c or sr25989 or zopya or zylagren or zyllt or ARC1779 or AZD6140 or alprostadil or asasantin or carnitine or cilostazol or clopidogrel or cloricromene or defibrotide or dilazep or dipyridamol* or disintegrin* or ditazol or E5880 or E5510 or epoprostenol* or fluribrofen or iloprost* or indobufen or isbogrel or ketanserin* or ketoprofen or ketorolac or levamisol* or ligustrazine* or tromethamine* or milrinone* or mopidamol* or naudicelle or nimesulide or ozagrel* or phthalzinol or picotamide or policosanol or prasugrel or procainamide or sarpogrelate or satigrel or sulphinpyrazone or sulfinpyrazone or suloctadil or terutroban or ticagrelor or ticlopidine or trapidil or triflusal or vorapaxar or oky046 or “oky 046” or oky 1581 or kbt3022 or kbt 3022 or fut 175 or cv4151 or cv 4151 or acylpyrin or aloxiprimum or colfarit or dispril or easprin or ecotrin or endosprin or magnecyl or micristin or polopirin or polopiryna or r16co5y76e or solprin or solupsan or zorprin):ti,ab,kw (Word variations have been searched)	34,114
#27	(ticlopidine* or ticagrelor* or abciximab* or eptifibatide* or tirofiban* or cilostazol* or dipyridamole* or (FX* adj4 (antag* or inhib* or block*)) or (Factor X* adj4 (antag* or inhib* or block*)) or dabigatran* or pradaxa or rendix or rivaroxaban or apixaban* or edoxaban* or betrixaban* or anisindione or apolate sodium or beciparcil or chlorophacinone or defibrotide or dextran sulfate or diphenadione or fluindione or ghilanten or glycerophosphoinositol inositolphosphodiesterase or glycosaminoglycan polysulfate or iliparcil or inclacumab or ljp 1082 or low molecular weight dextran sulfate or mopidamol or naroparcil or phenindione or ru 45,703 or tecarfarin or torapsel or tretoquinol or tretoquinol derivative or uproleselan or lomoparan or orgaran or apixaban or rivaroxaban or xarelto or edoxaban or lixiana or etexilate or agatroban or xabans):ti,ab,kw (Word variations have been searched)	12,496
#28	{OR #12–#27}	70,685
#29	#11 AND #28 with Publication Year from 2002 to 2023, with Cochrane Library publication date Between January 2002 and January 2023, in Trials	214

## Appendix B

Summary of the 28 records screened for inclusion/exclusion and the determining reasons behind each choice.

**Title**	**Include**	**Exclude**	**Explanation for Exclusion**
Kishore K, Hariprasad SM, Mungee S. Perioperative Antiplatelet Agents and Anticoagulants in Vitreoretinal Surgery. Ophthalmic Surg Lasers Imaging Retina. 2022 Feb;53(2):71-78. doi: 10.3928/23258160-20220128-01. Epub 2022 Feb 1. PMID: 35148217.		It is not a properly powered clinical study	Small case series
Guise PA, Wang K. Haemorrhagic complications following cataract and vitreoretinal surgery with sub-Tenon’s block in patients receiving non-vitamin K oral anticoagulant agents: A prospective audit. Anaesth Intensive Care. 2022 Jul;50(4):289-294. doi: 10.1177/0310057X211057136. Epub 2022 Jan 25. PMID: 35078342.	1. Included		
Starr MR, Ammar MJ, Patel LG, Boucher N, Yonekawa Y, Garg SJ, Hsu J, Ho AC, Regillo CD, Chiang A. Comparative Incidence of Postoperative Hemorrhage in Vitreoretinal Surgery in Patients on Anti-Coagulants. Ophthalmic Surg Lasers Imaging Retina. 2021 Jul;52(7):374-379. doi: 10.3928/23258160-20210628-03. Epub 2021 Jul 1. PMID: 34309424.	2. Included		
Lauermann P, Klingelhöfer A, Mielke D, van Oterendorp C, Hoerauf H, Striebe NA, Storch MW, Pfeiffer S, Koscielny J, Sucker C, Bemme S, Feltgen N. Risk Factors for Severe Bleeding Complications in Vitreoretinal Surgery and the Role of Antiplatelet or Anticoagulant Agents. Ophthalmol Retina. 2021 Aug;5(8):e23-e29. doi: 10.1016/j.oret.2021.04.013. Epub 2021 Apr 26. PMID: 33915331.	3. Included		
Louison S, Gabrielle PH, Soudry A, Meillon C, Blanc J, Béal G, Arsène S, Le Mer Y, Berrod JP, Kodjikian L, Creuzot-Garcher C; CFCR Research net. Perioperative risk of bleeding with antithrombotic agents in macular surgery: a national, prospective, multicentre study. Acta Ophthalmol. 2020 Dec;98(8):e991-e997. doi: 10.1111/aos.14434. Epub 2020 Apr 12. PMID: 32279459.	4. Included		
Bemme S, Lauermann P, Striebe NA, Khattab MH, Affeldt J, Callizo J, Bertelmann T, Pfeiffer S, Hoerauf H, Feltgen N. Risk of perioperative bleeding complications in rhegmatogenous retinal detachment surgery: a retrospective single-center study. Graefes Arch Clin Exp Ophthalmol. 2020 May;258(5):961-969. doi: 10.1007/s00417-019-04554-1. Epub 2020 Jan 7. PMID: 31907644.	5. Included		
Andonegui J, Capdevila F, Zubicoa A, Ibáñez B. Randomised controlled trial on vitreoretinal surgery with and without oral anticoagulants: surgical complications, visual results and perioperative thromboembolic events. Trials. 2019 Dec 4;20(1):677. doi: 10.1186/s13063-019-3805-6. PMID: 31801597; PMCID: PMC6894279.	6. Included		
Meillon C, Gabrielle PH, Luu M, Aho-Glele LS, Bron AM, Creuzot-Garcher C; CFSR research net. Antiplatelet and anticoagulant agents in vitreoretinal surgery: a prospective multicenter study involving 804 patients. Graefes Arch Clin Exp Ophthalmol. 2018 Mar;256(3):461-467. doi: 10.1007/s00417-017-3897-1. Epub 2018 Jan 23. PMID: 29362869.	7. Included		
Grzybowski A, Kanclerz P. Antiplatelet and anticoagulant agents in vitreoretinal surgery: a prospective multicenter study involving 804 patients. Graefes Arch Clin Exp Ophthalmol. 2018 Jul;256(7):1357. doi: 10.1007/s00417-018-3968-y. Epub 2018 Mar 30. PMID: 29602962; PMCID: PMC6006234.		Excluded	Comment
Ronald A Cantrell, Jeffrey Ryuta Willis, Susanna S Park, Yifeng Chia, Mariam Abouhossein, Giulio Barteselli; Post-vitrectomy ocular hemorrhage among United States adults on oral antithrombotics. Invest. Ophthalmol. Vis. Sci. 2018;59(9):883.		Excluded	Abstract for a conference
Shieh WS, Sridhar J, Hong BK, Maguire JI, Rahimy E, Shahlaee A, Ho AC, Hsu J, Regillo CD, Chiang A. Ophthalmic Complications Associated with Direct Oral Anticoagulant Medications. Semin Ophthalmol. 2017;32(5):614-619. doi: 10.3109/08820538.2016.1139738. Epub 2016 Jul 1. PMID: 27367495.		Excluded	Small case series, 6 patients
Mantopoulos D, Vavvas DG, Fine HF. Perioperative Risks of Antiplatelet and Anticoagulant Drugs in Vitreoretinal Procedures. Ophthalmic Surg Lasers Imaging Retina. 2017 Jan 1;48(1):4-8. doi: 10.3928/23258160-20161219-01. PMID: 28060388.		Excluded	Review
Ajudani, R., Khosravi, M. H., Ramezani-Binabaj, M., Rezaee-Zavareh, M. S., & Alishiri, A. agha. (2017). Cessation or Continuation of Aspirin in Patients Undergoing 20-gauge Pars Plana Vitrectomy due to Diabetic Retinopathy. Galen Medical Journal, 6(2), 95-101. https://doi.org/10.31661/gmj.v6i2.763	8. Included		
Grand MG, Walia HS. HEMORRHAGIC RISK OF VITREORETINAL SURGERY IN PATIENTS MAINTAINED ON NOVEL ORAL ANTICOAGULANT THERAPY. Retina. 2016 Feb;36(2):299-304. doi: 10.1097/IAE.0000000000000783. PMID: 26447397.	9. Included		
Brillat E, Rouberol F, Palombi K, Quesada JL, Bernheim D, Albaladejo P, Aptel F, Romanet JP, Chiquet C. A case-control study to assess aspirin as a risk factor of bleeding in rhegmatogenous retinal detachment surgery. Graefes Arch Clin Exp Ophthalmol. 2015 Nov;253(11):1899-905. doi: 10.1007/s00417-014-2900-3. Epub 2015 Jan 11. PMID: 25576171.	10. Included		
Witmer MT, Cohen SM. Oral anticoagulation and the risk of vitreous hemorrhage and retinal tears in eyes with acute posterior vitreous detachment. Retina. 2013 Mar;33(3):621-6. doi: 10.1097/IAE.0b013e3182671006. PMID: 23108264.	11. Included		
Ryan, A., Saad, T., Kirwan, C., Keegan, D.J. and Acheson, R.W. (2013), Bleeding risks of vitreoretinal surgery. Clin Experiment Ophthalmol, 41: 387-395. https://doi-org.ezproxy.uio.no/10.1111/ceo.12017	12. Included		
Passemard M, Koehrer P, Juniot A, Bron AM, Creuzot-Garcher C. Maintenance of anticoagulant and antiplatelet agents for patients undergoing peribulbar anesthesia and vitreoretinal surgery. Retina. 2012 Oct;32(9):1868-73. doi: 10.1097/IAE.0b013e31825097ae. PMID: 22495328.	13. Included		
Malik AI, Foster RE, Correa ZM, Petersen MR, Miller DM, Riemann CD. Anatomical and visual results of transconjunctival sutureless vitrectomy using subconjunctival anesthesia performed on select patients taking anticoagulant and antiplatelet agents. Retina. 2012 May;32(5):905-11. doi: 10.1097/IAE.0b013e31822f55c4. PMID: 22298013.	14. Included		
Mason JO 3rd, Gupta SR, Compton CJ, Frederick PA, Neimkin MG, Hill ML, Heersink MJ, Vail RS, White MF Jr, Feist RM, Thomley ML, Albert MA Jr. Comparison of hemorrhagic complications of warfarin and clopidogrel bisulfate in 25-gauge vitrectomy versus a control group. Ophthalmology. 2011 Mar;118(3):543-7. doi: 10.1016/j.ophtha.2010.07.005. Epub 2010 Sep 29. PMID: 20884061.	15. Included		
Fabinyi DC, O’Neill EC, Connell PP, Clark JB. Vitreous cavity haemorrhage post-vitrectomy for diabetic eye disease: the effect of perioperative anticoagulation and antiplatelet agents. Clin Exp Ophthalmol. 2011 Dec;39(9):878-84. doi: 10.1111/j.1442-9071.2011.02575.x. Epub 2011 Jun 14. PMID: 21631675.	16. Included		
El-Sanhouri AA, Foster RE, Petersen MR, Hutchins RK, Miller DM, Evans TM, Trichopoulos N, Riemann CD. Retinal tears after posterior vitreous detachment and vitreous hemorrhage in patients on systemic anticoagulants. Eye (Lond). 2011 Aug;25(8):1016-9. doi: 10.1038/eye.2011.106. Epub 2011 May 13. PMID: 21587275; PMCID: PMC3178220.	17. Included		
Chandra A, Jazayeri F, Williamson TH. Warfarin in vitreoretinal surgery: a case controlled series. Br J Ophthalmol. 2011 Jul;95(7):976-8. doi: 10.1136/bjo.2010.187526. Epub 2010 Nov 11. PMID: 21071761.	18. Included		
Brown JS, Mahmoud TH. Anticoagulation and clinically significant postoperative vitreous hemorrhage in diabetic vitrectomy. Retina. 2011 Nov;31(10):1983-7. doi: 10.1097/IAE.0b013e31821800cd. PMID: 21836531.	19. Included		
Fu AD, McDonald HR, Williams DF, Cantrill HL, Ryan EH Jr, Johnson RN, Ai E, Jumper JM. Anticoagulation with warfarin in vitreoretinal surgery. Retina. 2007 Mar;27(3):290-5. doi: 10.1097/01.iae.0000243033.39301.10. PMID: 17460583.	20. Included		
Dayani PN, Grand MG. Maintenance of warfarin anticoagulation for patients undergoing vitreoretinal surgery. Trans Am Ophthalmol Soc. 2006;104:149-60. PMID: 17471335; PMCID: PMC1809899.	21. Included		
Narendran N, Williamson TH. The effects of aspirin and warfarin therapy on haemorrhage in vitreoretinal surgery. Acta Ophthalmol Scand. 2003 Feb;81(1):38-40. doi: 10.1034/j.1600-0420.2003.00020.x. PMID: 12631017.	22. Included		
